# The medial prefrontal cortex to the medial amygdala connections may affect the anxiety level in aged rats

**DOI:** 10.1002/brb3.2616

**Published:** 2022-05-23

**Authors:** Narges Sotoudeh, Mohammad Reza Namavar, Farshid Bagheri, Asadollah Zarifkar

**Affiliations:** ^1^ Department of Anatomical Sciences School of Medicine Shiraz University of Medical Sciences Shiraz Iran; ^2^ Histomorphometry and Stereology Research Center Shiraz University of Medical Sciences Shiraz Iran; ^3^ Clinical Neurology Research Center Shiraz University of Medical Sciences Shiraz Iran; ^4^ Department of Physiology School of Medicine Shiraz University of Medical Sciences Shiraz Iran; ^5^ Shiraz Neuroscience Research Center Shiraz University of Medical Sciences Shiraz Iran

**Keywords:** aging, anxiety, mPFC–amygdala connection, sex difference

## Abstract

**Background:**

Aging changes brain function and behavior differently in male and female individuals. Changes in the medial prefrontal cortex (mPFC)–medial amygdala (MeA) connectivity affect anxiety‐like behavior.

**Objectives:**

Therefore, this study aimed to investigate the effect of aging and sex on the mPFC–MeA connection and its association with the level of anxiety‐like behavior.

**Methods:**

We divided the Wistar rats into the male and female young rats (2‐3‐month‐old) and male and female old rats (18–20 months old). First, the open field test (OFT) was performed, and then 80 nl of Fluoro‐Gold (FG) was injected by stereotaxic surgery in the right or left MeA. After 10 days, the animals were perfused, their brain removed, coronal sections cut, and the number of FG‐labeled cells in the right and left mPFC of each sample was estimated.

**Results:**

Based on our results, old animals revealed less anxiety‐like behavior than young ones, and young females were less anxious than young males, too. Interestingly, MeA of old male rats received more fibers from the bilateral mPFC than young ones. Also, this connection was stronger in the young females than young males. Altogether, the present study indicated that old individuals had less anxiety‐like behavior and stronger mPFC–MeA connection, and young female rats were less anxious and had a stronger connection of mPFC–amygdala than males of the same age.

**Conclusion:**

Thus, it seems that there is a negative relationship between anxiety levels based on the rat's performance in the OFT apparatus and the mPFC–MeA connection.

## Graphical abstract

Old animals have less anxiety‐like behavior and stronger mPFC‐ medial amygdala connection than young ones.

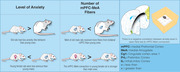

## 1 INTRODUCTION

Aging is considered to be a gradual and progressive alteration in brain function and behavioral presentation over a life (Shoji & Miyakawa, 2019). An association between typical brain aging and cognitive decline even in the absence of neurodegeneration has been reported (Grady, 2012). The neuroanatomical basis of this age‐related functional impairment is yet to be well known. Changes in behavior, especially its emotional aspect, represent one of the essential features of aging in humans and animals (Ebner & Fischer, 2014). In recent years, several studies have reported changes in the anxiety level with age, as evaluated by the performance of rats in behavioral models of anxiety for example the open‐field test (Boguszewski & Zagrodzka, 2002; Torras‐Garcia et al., 2005); some studies have related the impairments in aged animals with changed structure and function of specific brain regions (Dickstein et al., 2007; Samson & Barnes, 2013).

The critical role of the amygdala and medial prefrontal cortex (mPFC) in behavioral circumstances that involve competition between bottom‐up and top‐down processes has been the target of numerous studies (Bishop, 2007; Ochsner & Gross, 2005; Quirk & Beer, 2006). It is believed that the mPFC has a role in the regulation and control of amygdala output and the accompanying behavioral phenomena (Ochsner & Gross, 2005). More recent studies have proposed that the structural and functional connectivity between these two regions is a better predictor of these outcomes than the activity of either region alone; also, because of the strong reciprocal connections between the amygdala and the mPFC, it seems that both regions need to be investigated as one circuit (Kim et al., 2011; Pezawas et al., 2005).

The idea here is that the stronger connection between these two regions may have resulted in a better behavioral outcome in terms of reported anxiety. Whereas several studies have demonstrated a negative correlation between trait anxiety and structural integrity of the amygdala‐PFC pathway (Baur et al., 2012; Kim & Whalen, 2009), other studies have in contrast reported a positive association between trait anxiety and this connection (Modi et al., 2013; Montag et al., 2012). It has been proposed that a changed balance between activity in the amygdala and PFC could be a neural mechanism underlying anxiety (Bishop, 2007). Converging findings from animal and human research suggest that anxiety‐related behaviors involve top‐down regulatory influences from the PFC to the amygdala (Banks et al., 2007; Ghashghaei et al., 2007), and successful emotion regulation is the concern with lower amygdala activity and higher PFC activity (Phelps et al., 2004; Wager et al., 2008). The mPFC regulates the output of the amygdala to control anxiety, so less structural connectivity may disable top‐down control over the amygdala and, consequently, leads to an increase in anxiety. In the present study, we will focus on the top‐down connectivity between the mPFC and the medial amygdala (MeA). Most of the prefrontal–amygdala connections are focused on the basolateral amygdala (BLA), as opposed to the central nucleus amygdala (Ce). Based on animal studies of fear conditioning and extinction, mPFC input to the BLA is responsible for inhibiting amygdala output by regulating BLA inputs to the Ce. Among the amygdaloidal structures involved in chemosensory‐mediated emotional behavior, the MeA plays a central role in generating innate emotional responses to chemo‐signal, has high susceptibility to aging, and contains cells that express substance P that regulates the anxiety process (Brennan & Zufall, 2006; LeDoux, 2012; Olucha‐Bordonau et al., 2015). It is a subcortical structure and is probably an output station, controlled by its cortical inputs (Cádiz‐Moretti et al., 2016). Thus, it is noticeable that little is known about the MeA functional connectivity.

Importantly, some investigators have suggested that age‐related differences in functional connectivity may be different depending upon task demands; for instance, in an fMRI study, older adults presented enhanced functional connectivity between the prefrontal cortex and the amygdala during successful encoding of positive emotion relative to younger adults (Addis et al., 2010). In contrast, St. Jacques et al. (2009) reported a decline in the functional connectivity during the encoding of negative information. In another study, Arruda‐Carvalho et al. examined a relatively broad window of development (six‐time points from P10 to P80) and reported massive mPFC–BLA fiber proliferation between P10 and P30. They confirmed a modest decrease later in adolescence between P45 and adulthood in mice (Arruda‐Carvalho et al., 2017). The relationship between the structure of this connection and anxiety has not been directly studied in aging. Furthermore, recent fMRI studies have stated sex‐related hemispheric lateralization of amygdala involvement in memory of emotional arousing; the right amygdala was linked with more functional connectivity in men than in women. In contrast, the left amygdala was associated with greater functional connectivity in women (Kilpatrick et al., 2006). Wager et al. (2003) also report that in emotion studies, particularly for negative emotions, amygdala activations are lateralized to the left. Results of the meta‐analysis also revealed that the left amygdala activation is pointed out in more studies than the right amygdala (Baas et al., 2004). However, the role of lateralization of mPFC–MeA connection and sex differences is unclear. Knowledge associated with hemispheric differences could be supportive in our understanding of the neural basis of emotion processing. Individual emotion studies that report lateralization in amygdala activation use varying paradigms and are limited by statistical power and sensitivity; therefore, there is still uncertainty in this area of study (Baas et al., 2004). We nevertheless do not know whether the right or left amygdala is more consistently involved in the anxiety process, and there is a lack of knowledge on the effect of age and sex on the level of anxiety. The present study aimed to investigate whether changes in the mPFC–MeA connection can indeed be observed in the old rats and coincide with changes in anxiety behavior in the behavioral models of anxiety, the open field test; the effect of gender, and hemisphere lateralization in this connection and anxiety level was also assessed.

## 2 MATERIALS AND METHODS

### 2.1 Animals

Eighty Wistar rats including the male (*n* = 20) and female (*n* = 20) young rats (2–3 month old), and male (*n* = 20) and female (*n* = 20) old rats (18–20 month old) were purchased from the Institute of Animal Science Laboratory of Tehran (Medzist, Tehran, Iran). Animals were maintained under a standard 12–12 h light–dark cycle with lights on at 7:00 a.m. at room temperature (25 ± 2°C). Food and water were available ad libitum. All procedures were performed according to the NIH Guide for the care and use of laboratory animals and the Animal Research: Reporting in Vivo Experiments (ARRIVE) guiding principle and approved by the Ethics Committee of Shiraz University of Medical Sciences (SUMS, Shiraz, Iran, Ethic code: IR.SUMS.REC.1397.77).

### 2.2 Evaluation of anxiety using OFT

The open‐field test (OFT) is an experimental test used to evaluate the general locomotor activity level and anxiety of rodents in scientific research. Animals such as rats and mice display a natural dislike to brightly lit open areas. However, they also have the drive to explore an observed threatening stimulus. Decreased levels of anxiety lead to increased exploratory behavior, more locomotion, and a preference to stay in the center of the field (Sestakova et al., 2013). The open‐field arena consists of a transparent Plexiglas cube (100 × 100 × 50 cm), and it was divided into a central zone and an outer zone in the periphery. OFT is assessed with EthoVision‐XT video tracking (Noldus Information Technology Inc., Netherlands). On the day before surgery, the room test was prepared, and the camera and software were adjusted. The animal was placed in the center of the cube for 15 min, and animal activity was recorded. The software measured and analyzed the total time spent in the center zone (s), latency time for the first entrance to the center zone (s), and total traveled distance (cm) over 15 min. At the end of the test, the rat was removed from the maze, and fecal boli were counted visually.

### 2.3 Fluoro‐Gold injections in the right and left medial amygdala

The animals were anesthetized with an intraperitoneal injection of ketamine (100 mg/kg) and xylazine (10 mg/kg). The heads were fixed in a stereotactic frame (David Kopf Instruments, California, USA). A skin incision was made to expose the skull, a drill hole was made according to Paxinos atlas, a stainless steel guide cannula (22‐gauge) was implanted into the amygdala (AP = −2, ML ± 4.8, DV − 8.6), and 80 nl of Fluoro‐Gold 2% (FG; Fluorochrome, Denver, Colorado, USA) was injected in the MeA (Right/Left) within 10 min (Gruene et al., 2015; Vafaee et al., 2018).

### 2.4 Tissue processing and histology

After 10–12 days of surgery, the rats were transcardially perfused with PBS, followed by 200–300 ml of 4% paraformaldehyde in 0.1 M phosphate buffer, pH 7.4. The brain was rapidly removed and post‐fixed in the same fixative overnight. It was then transferred to a 30% sucrose solution in 0.1 M PBS, 4°C for 48–72 h. Samples were then kept at −80°C. Thick coronal sections (50 μm) of the brains were cut by cryostat (Leica CM1860, Germany) and mounted on gelatin‐coated slides. It has to be noted that the tissues and sections were kept in the darkness in all steps.

### 2.5 Quantification and mapping of cells retrogradely labeled with Fluoro‐Gold

The sections were imaged on a fluorescence microscope using a 20× objective (Nikon, Japan), using Zeiss ZEN Imaging Software (Zeiss, Germany). Tiled digital images were captured for each region of interest (mPFC parts) and analyzed offline, and the number of positively labeled cells was counted. For the purpose of description, projections to the MeA from the mPFC area (Cg, Pl, and Il) were considered to be “very strong” (++++; >300 cells/nuclei/animal), “strong” (+++; 200–300), and “moderate” (++; 100–200) or “weak” (+; 50–100) (Qi et al., 2009). Finally, based on the average results of neuron distribution and number in each animal group, the schematic diagram of coronal sections throughout the mPFC was drawn.

### 2.6 Statistical analysis

All behavioral tests and decoding were performed blindly, and behavioral data were analyzed using software Prism 6 for Windows (GraphPad Software Inc., USA). First, the Shapiro test was used to determine the normal distribution of data. Second, the data were analyzed with two‐way ANOVA, followed by post hoc Tukey's HSD test. Data are reported as the mean ± SD, and the statistical significance level in all tests was considered *p*< .05.

To determine the correlation between anxiety parameters and the number of afferents fibers of MeA (right/left) from the mPFC sub‐regions, we used Pearson and Spearman tests for parametric and nonparametric data, respectively.

## 3 RESULTS

### 3.1 Behavioral test: OFT

**FIGURE 1:**
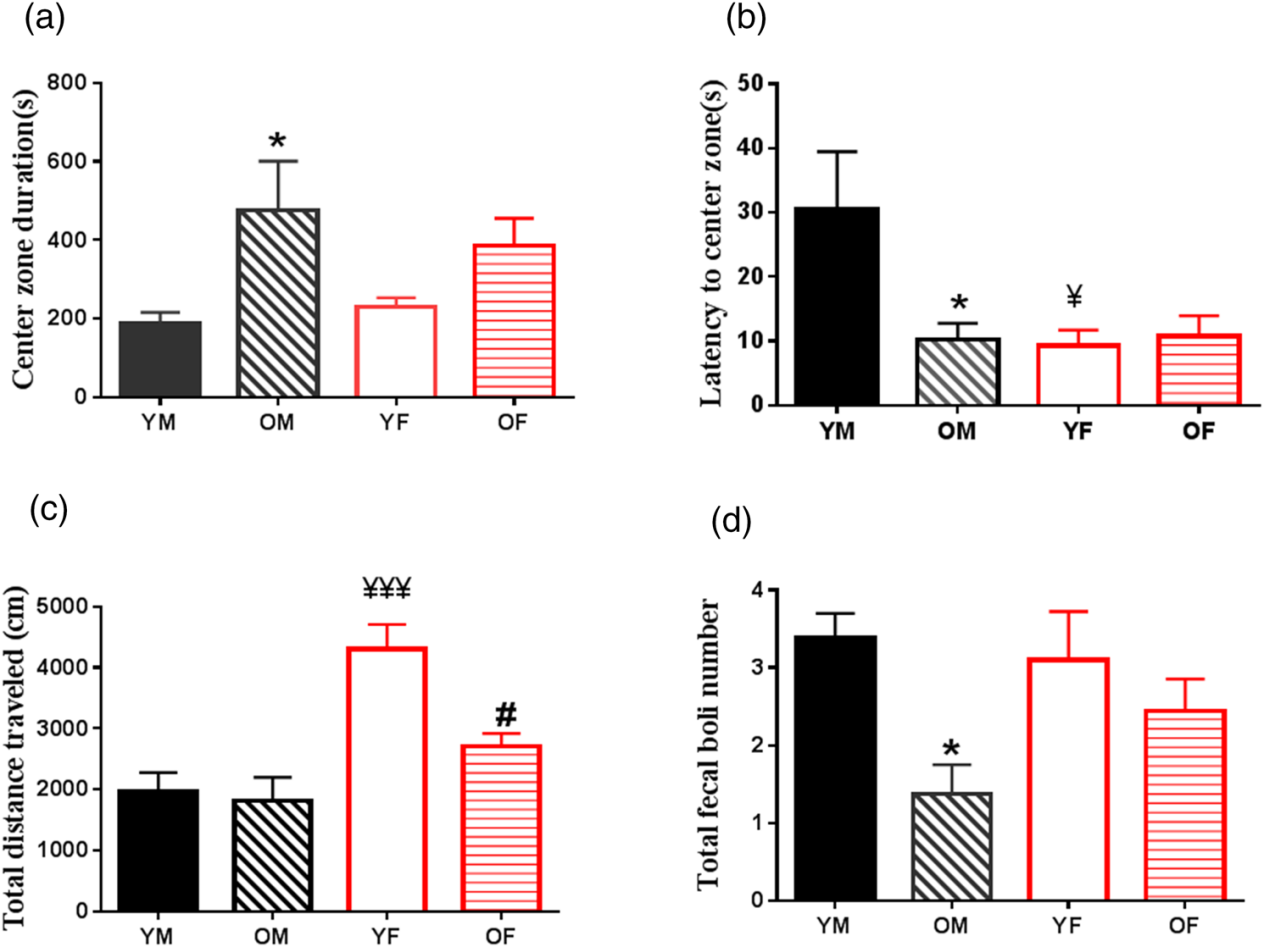
The activity of rats in the open field test: Mean ± standard deviation of time spent on the center zone (a), the latency of the first entrance to the center zone (b), total distance traveled (c), and total fecal boli count (d) in the young (YM) and old male (OM) and young (YF) and old (OF) female rats. ^*^ *p*<.05 vs. YM**; ^#^** *p*<.5 vs. YF; ^¥^ *p*<.05; ^¥¥¥^ *p*<.001 vs. YM

The anxious behaviors of animals in all groups were assessed on the day before surgery, and the parameters of latency to center zone (seconds), center zone duration (seconds), traveled distance (cm), and fecal boli (number) were estimated within 15 min. Based on the final results from EthoVision‐XT video tracking, the time that animals spent in the center zone was higher in both old groups in comparison with the young groups, but this difference was significant in the old male (OM) group (*p*<.05, Figure 1a). There was no significant sex difference between the young and old groups. The latency time of the first entrance to the center zone in the OM group was significantly shorter in comparison with the young male (YM) rats. This factor was shorter in the YF group in comparison with the YM group (*p*<.05, Figure 1b). The traveled distance in the old female (OF) rats in comparison with the young females (YF) significantly decreased (*p*<.05), while this parameter in YF animals in comparison with YM increased (*p*<.001, Figure 1c).

In addition, both parameters of latency to center zone and traveled distance showed significant age–sex interaction (*p*<.02).

Counting the number of defecations showed that aging reduced this parameter in both males and females. However, this difference was only statistically significant in the male groups (*p*<.05, Figure 1d). In addition, there was no significant sex difference between the young and old groups in this parameter.

### 3.2 Determination and confirmation of the injection site

**FIGURE 2:**
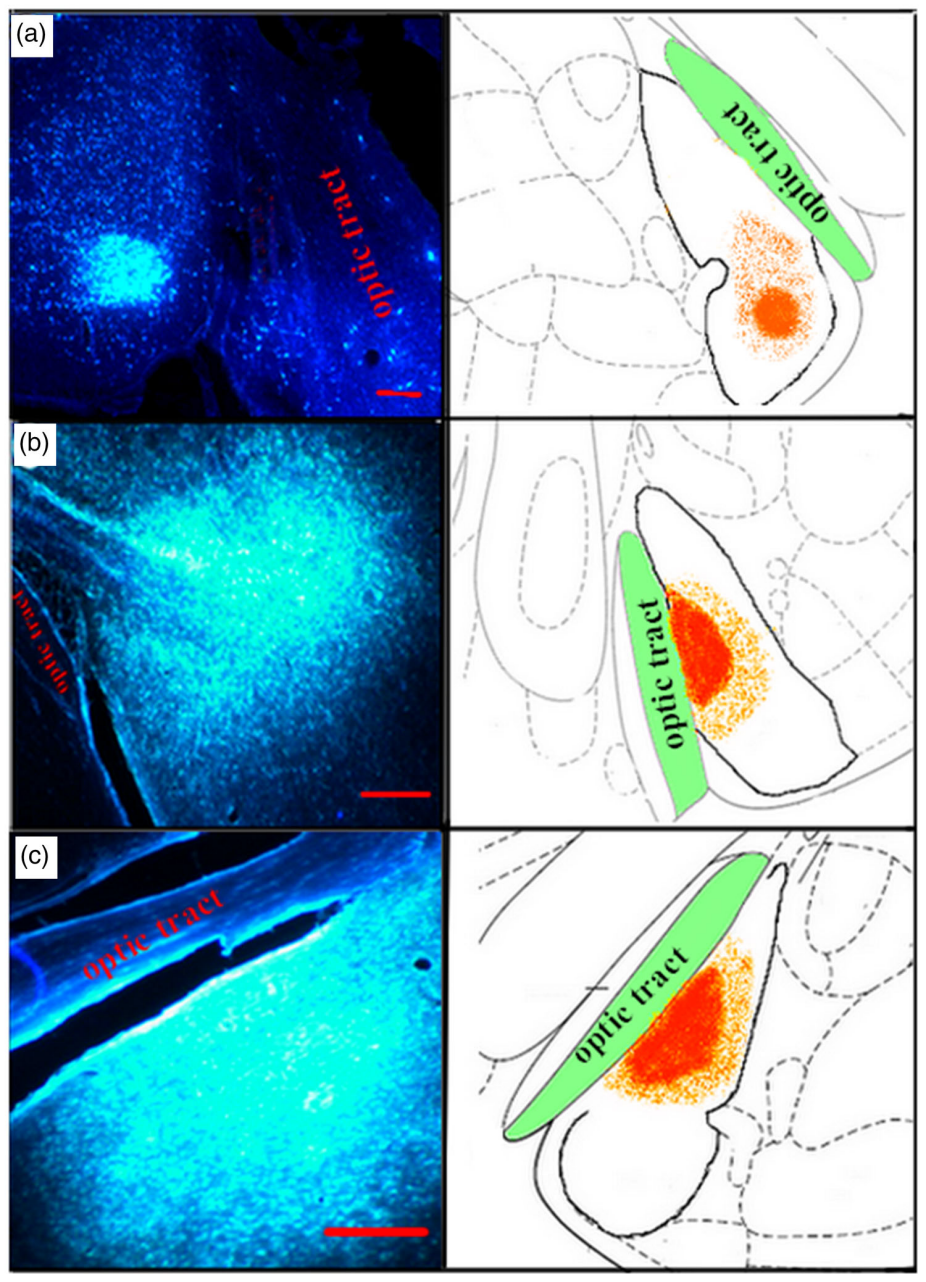
Representative microphotograph and schematic of the injection site of Fluoro‐Gold in the medial amygdala (MeA) region and its schematic diagram: (a) right‐MeA (AP = −2.64, ML = 3.8, DV = 9.2), (b) left‐MeA (AP = −3, ML = 4.0, DV = 9.0, and (c) left‐MeA (AP = −3.12, ML = 4, DV = 9.2) in the experimental groups. Red plot indicates the extent of Fluoro‐Gold injection site. Scale bar = 100 μm

Injections of Fluoro‐Gold were made into the MeA (*n* = 10) of the right or left hemisphere. Firstly, the injection site was checked in all animals; if it was not located in the central or dorsal MeA, it was excluded. Ultimately, seven cases were selected for the study of afferent fibers from the cingulate (Cg), prelimbic (Pl), and infralimbic (Il) regions of the mPFC for each hemisphere. Figure 2 shows some examples of injection sites in different coordination.

### 3.3 Afferents to MeA

**FIGURE 3:**
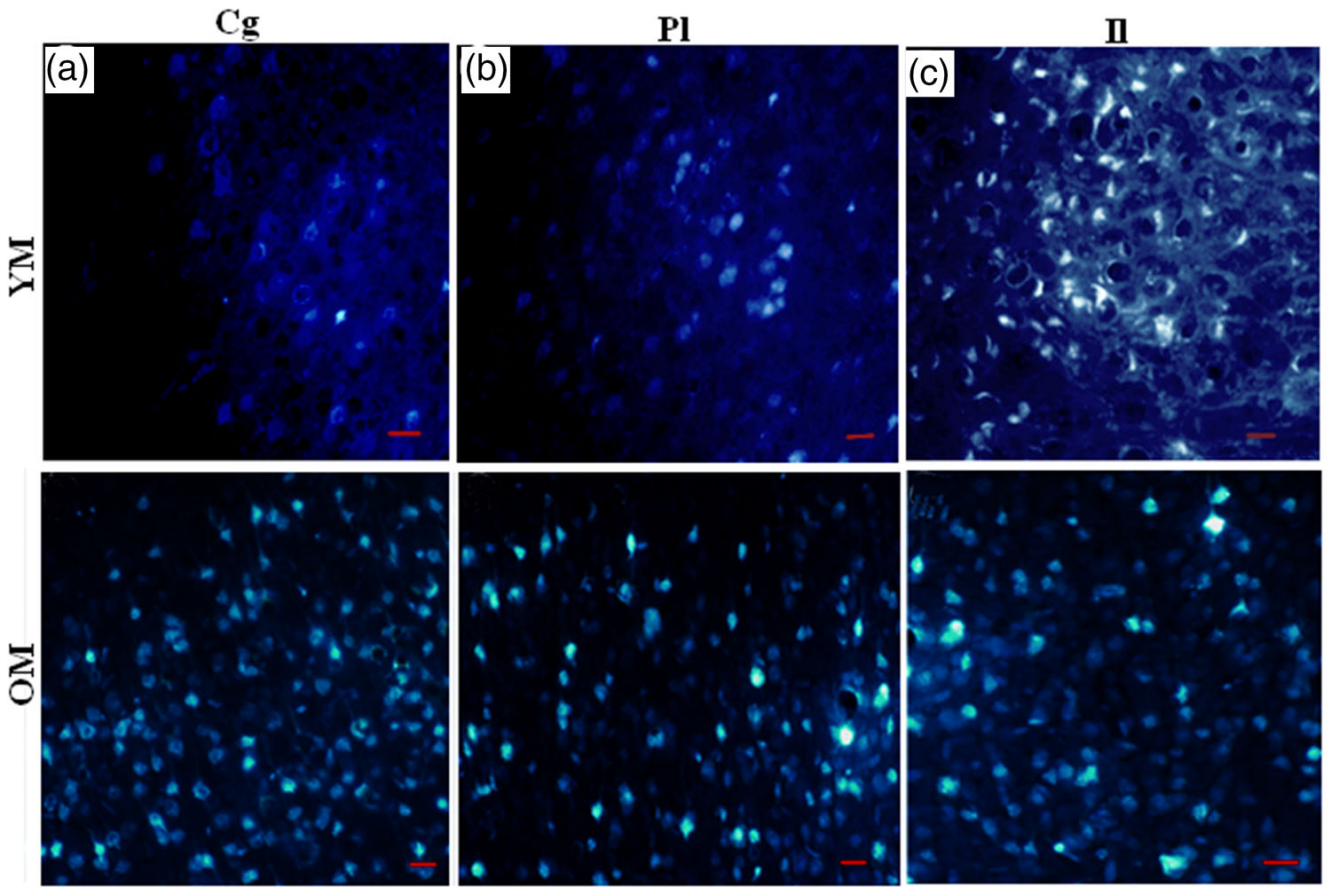
Photomicrograph samples of the retrograde‐labeled cells in the subdivisions of the medial prefrontal cortex (cingulate, prelimbic, and infralimbic) after injection of Fluoro‐Gold in the medial amygdala; (a) cingulate (cg), (b) prelimbic (Pl), and (c) infralimbic (Il) regions in the young male (YM) group in the top row and old male (OM) group in the bottom row. Scale bar = 30 μm

The definition and description of the medial prefrontal cortical areas follow the scheme suggested by Krettek and Price (1977); the infralimbic area includes the band of cortex characterized by poor lamination and an uneven lamina II, the prelimbic area lies dorsal to the infralimbic area and is conspicuous by its densely packed lamina VI, and the anterior cingulate areas lie dorsal to the prelimbic area and can be distinguished from the latter by their greater depth and the looser arrangement of constituent cells (Cassell & Wright, 1986). Figure 3 presents an example of the retrogradely labeled cells in the subdivision of the medial prefrontal cortex (cingulate, prelimbic, and infralimbic) after injection of Fluoro‐Gold in the medial amygdala.

#### 3.3.1 Retrograde labeled neurons in the mPFC subregions after injection of FG into the R‐MeA

**FIGURE 4:**
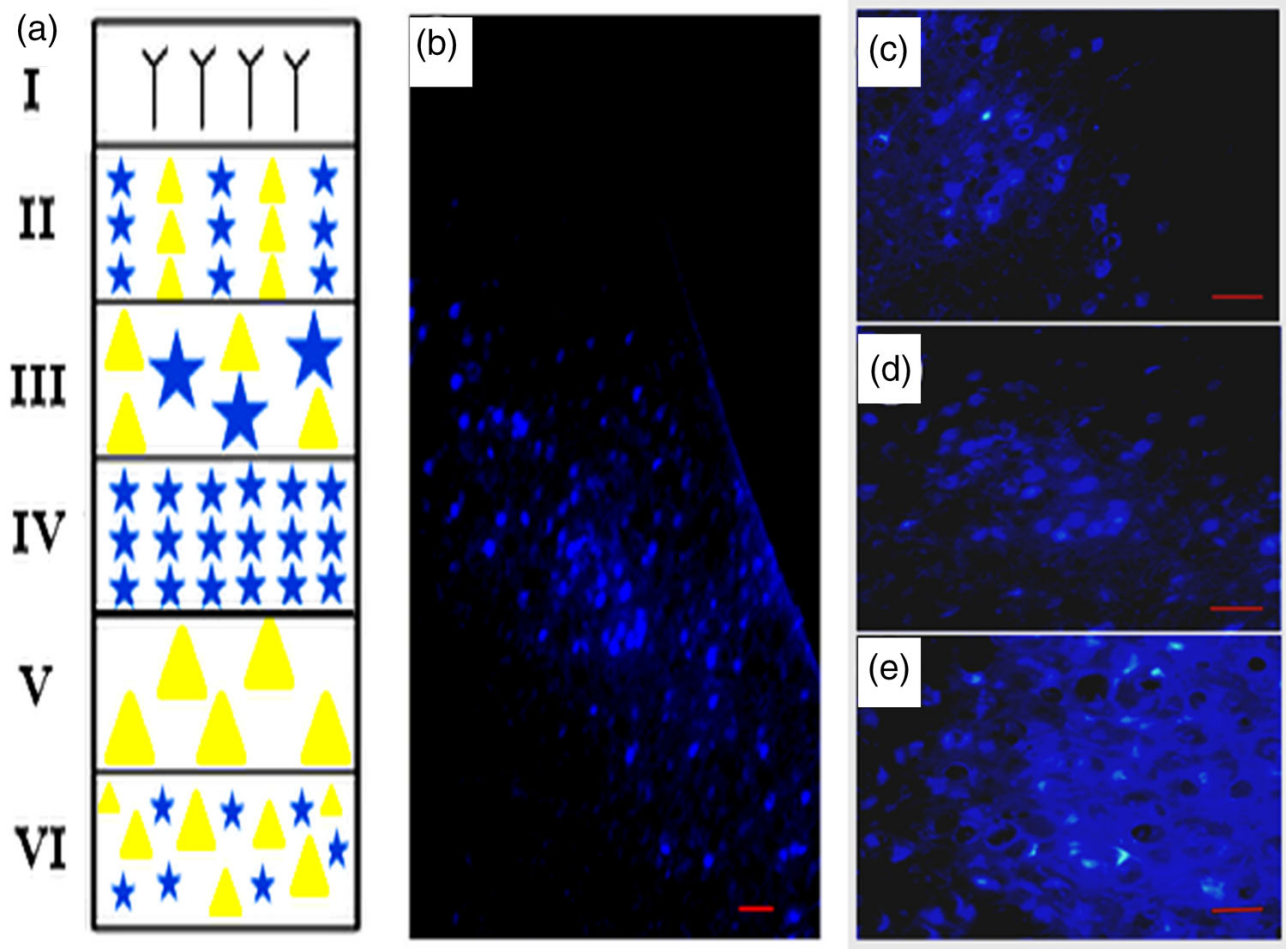
(a) Schematic of different cell layers of the brain cortex (layers I, II, III, IV, V, and VI). (b) Photomicrograph of distribution of Fluoro‐Gold‐positive cells in the medial prefrontal cortex subregions, (c) magnification of positive cells in deep layers of cingulate, (d) different layers of prelimbic, and (e) infralimbic areas. Scale bar = 60 μm

The positively stained cells were observed bilaterally (Cg, Pl, and Il subregions) in all four groups of YM, YF, OM, and OF:

Young male group: The labeled cells were found moderate (++) in Pl, weak (+) in both Cg, and Il areas ipsilaterally and moderate in Cg and Pl, and weak in Il areas contralaterally (Table 1). The labeled neurons were predominantly located in deep layers of the cingulate region, whereas these labeled neurons are scattered in prelimbic and infralimbic regions in all cortical layers, as shown in Figure 4.

**TABLE 1:** Distribution of Fluoro‐Gold‐labeled neurons throughout the cingulate (Cg), prelimbic (Pl), and infralimbic (Il) subregions of the medial prefrontal cortex after injection of Fluoro‐Gold in the right medial amygdala in experimental groups

	R‐hemisphere			L‐hemisphere		
Groups	Cg	Pl	Il	Cg	Pl	Il
YM	64^+^ ±19	151^++^±83	92**^+^** ±37	131^++^ ±71	153**^++^** ±59	110^++^±47
YF	637^++++^±188	514^++++^±78	213^+++^±96	347^++++^±56	464^++++^±97	147^++^±97
OM	356^++++^±170	282^+++^±163	156^++^±91	388^++++^±90	309^++++^±185	159^++^±98
OF	508^++++^±97	247^+++^±100	164^++^±87	402^++++^±106	326^++++^±87	187^++^±51

Abbreviations: **++++**, very strong; **+++**, strong; **++**, moderate; **+**, weak; OF, old female; OM, old male; YF, young Female; YM, young male.

Young female group: The R‐MeA received fibers from the bilateral Cg and Pl areas very strongly, ipsilateral Il strongly, and contralateral Il moderately (Table 1).

Old male group: Bilateral Cg and Pl regions send fibers to R‐MeA very strongly, while Il regions of both sides retained a moderate connection with the R‐MeA (Table 1).

Old female group: On the ipsilateral side, Cg, Pl, and Il had very strongly, strongly, and moderately connections with the R‐MeA, respectively, whereas this connection was observed very strong in Cg and Pl, and moderate in Il contralaterally (Table 1).

Age differences: The R‐MeA of old rats, particularly the male group, received more afferent fibers from the right and left mPFC subregions than young ones. These labeled neurons stained more intensely in areas of Cg, Pl, and Il as well (Table 1 and Figure 3).

**FIGURE 5:**
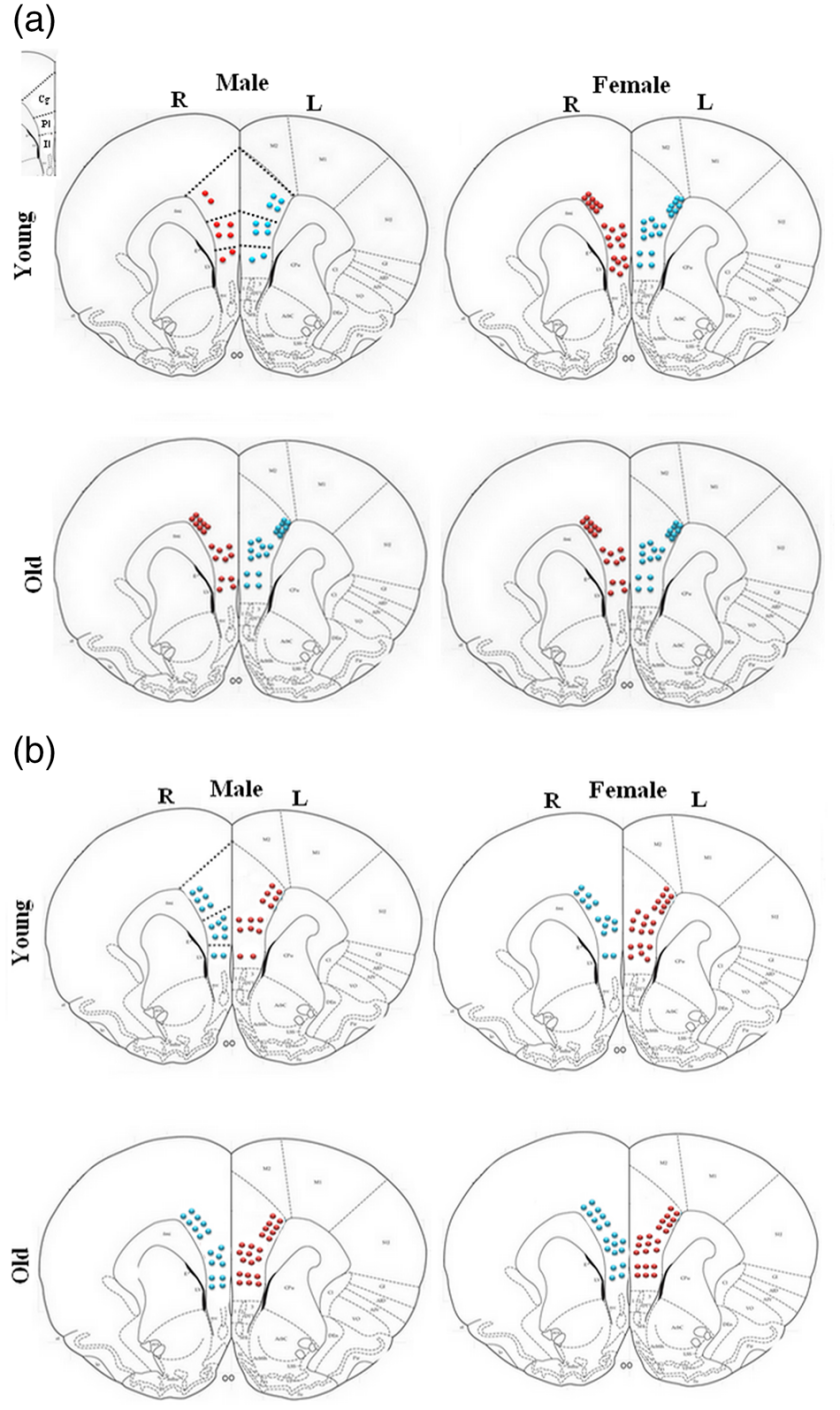
Schematic diagrams of coronal sections throughout the mPFC subdivisions (Cg, Pl, and Il) detailing the distribution of retrogradely labeled neurons, ipsilateral, and contralateral to the injection site of Fluoro‐Gold into the right medial amygdala (R‐MeA) (a) and left medial amygdala (L‐MeA) (b) in the experimental groups. The dots have been designed based on the result in Tables 1 and 2 (two dots = +). Red dots show the ipsilateral positive labeled cells and blue dots show the contralateral positive labeled cells in mPFC subregions. Bregma = +2.52

Sex differences: Between young groups, the R‐MeA of the female group showed a stronger connection with the mPFC subdivisions than male ones, while all regions of Cg, Pl, and Il sent relatively equal fibers to the right MeA in both old groups (Figure 5).

Lateralization: In all groups of YM, OM, and OF, right and left mPFC subregions had an equal connection with R‐MeA, while in the YF group right Il had a stronger connection with R‐MeA than the left side.

#### 3.3.2 Retrograde labeled neurons in the mPFC regions after injection of FG into the L‐MeA

The bilateral connection was observed from all subregions of mPFC (Cg, Pl, and Il) to the L‐MeA in all groups:

Young male group: The labeled cells spread strongly (+++) in the deep layer of Cg and Pl areas and weak (+) in Il areas bilaterally (Table 2).

**TABLE 2:** Distribution of Fluoro‐Gold‐labeled neurons throughout the cingulate (Cg), prelimbic (Pl), and infralimbic (Il) subregions of the medial prefrontal cortex after injection of Fluoro‐Gold in the left medial amygdala in experimental groups

	R‐hemisphere			L‐hemisphere		
Groups	Cg	Pl	Il	Cg	Pl	Il
YM	239^+++^±89	328^+++^±76	99^+^±9	223^+++^±44	251^+++^±126	89^+^±33
YF	391^+++^±137	259^+++^±95	95^+^±25	329^++++^±107	358++++±89	288^+++^±36
OM	411^++++^±74	316^++++^±66	153^++^±31	569^++++^±73	386^++++^±35	236^+++^±44
OF	506^++++^±168	272^+++^±39	147^++^±41	646^++++^±100	434^++++^±193	214^+++^±70

Abbreviations: **++++**, very strong; **+++**, strong; **++**, moderate; **+**, weak; OF, old female; OM, old male; YF, young Female; YM, young male.

Young female group: The L‐MeA received fibers from contralateral Cg and Pl strongly and Il weakly, while the ipsilateral Cg and Pl projected fibers very strongly and Il strongly to L‐MeA (Table 2).

Old male group: On both sides, Cg and Pl sent fibers to the L‐MeA very strongly, while contralateral Il and ipsilateral Il sent these fibers to the L‐MeA strongly and moderately, respectively (Table 2).

Old female group: Positive cells were distributed very strongly in Cg, strongly in Pl, and moderately in Il regions of the contralateral side. On ipsilateral sides, Cg and Pl projected fibers very strongly and Il strongly to the L‐MeA region (Table 2).

Age differences: In both aged groups, afferent fibers from the Il region to L‐MeA were stronger than in young groups, while other regions had almost an equal connection.

Sex differences: The L‐MeA in the young female rats had a stronger connection with the mPFC than in male ones and in both young groups, and the L‐MeA received fibers from the right Il region. In the case of aged rats, the sole difference was the stronger connection of the right‐Pl area with the L‐MeA, while other regions had a quite equal connection.

Lateralization: In all groups of YM, OM, and OF, right and left mPFC subregions had an equal connection with L‐MeA, while in the YF group left infralimbic had a stronger connection with L‐MeA than the left side.

### 3.4 Mapping of cells retrogradely labeled with Fluoro‐Gold

Based on the final results of the number and distribution of Fluoro‐Gold‐labeled neurons in the mPFC subregions in the young and old groups, schematic diagrams through the coronal mPFC sections were drawn: the number of the colored blot in all three regions of cingulate, prelimbic, and infralimbic were designed based on the number of Fluoro‐Gold‐labeled neurons in these regions and every two plot shows 50–100 neurons, and they are distributed in deep layers of the cingulate area and all cortical layers of prelimbic and infralimbic areas. In the case of the number of labeled neurons, both old male and young female groups had more positive cells in all mPFC subregions bilaterally when compared to their same age groups. The red plot shows the Fluoro‐Gold‐labeled neurons in the ipsilateral hemisphere, and the blue plot shows the labeled neurons on the contralateral side after injection of FG to MeA (Figure 5).

### 3.5 The correlation between the anxiety‐like behaviors and the mPFC–MeA connection

For the first time, the correlation between anxiety behavior and anatomical connections between these two regions is presented; in the old male group, the center zone duration and the distance traveled had a significant positive correlation with the number of efferent fibers from the mPFC to the MeA (*p* = .02, *r* = .8). In other groups, there was no significant correlation between anxiety factors and the connections between these two areas. Like so, it means that less anxiety may be associated with a stronger top‐down connection of the mPFC‐MeA in the old male Wistar rats.

## 4 DISCUSSION

Typical brain aging is associated with a weakening in cognitive functions (Grady, 2012) and increases the risk of mental health disorders such as anxiety; one pathway by which adversity may confer anxiety‐behavior is through changes in the connections between the amygdala (MeA) (Phelps & LeDoux, 2005) and medial prefrontal cortex (mPFC), two brain regions which are thought to modulate emotion expression and regulation (McLaughlin et al., 2014). Because the MeA and mPFC perform critical roles in the expression of emotions (Bishop, 2007), disruptions in the mPFC–MeA functional connectivity could be related to the possible changes in the anxiety level. Therefore, in the current study, we evaluated the top‐down connection of the mPFC–MeA by injection of the retrograde tracer, Fluoro‐Gold, in the right or left MeA and anxiety level by the open field test in the young and aged male and female Wistar rats.

To the best of our knowledge, the anatomical connection of mPFC–MeA in old animals or humans is not completely understood. However, some studies have described changes in the performance of aged animals in the OFT. Based on the results of the OFT test in the case of aging in the present study, old male animals showed less anxiety‐like behavior than young male rats; shorter latency time to center zone and fecal boli count, and spent more time in the center zone.

A previous study stated that the less anxious mice tended to enter the center zone more than anxious ones and stayed there longer, and also, the defecation was less (Seibenhener & Wooten, 2015). In agreement with our results, Shoji and Miyakawa (2019) have also reported that there is an increase in the time spent in the center area in this test in old mice (46 weeks) when compared with the young ones (7 weeks). Also, in another research, male and female spontaneously hypertensive rats (SHR), Wistar–Kyoto (WKY), and Sprague–Dawley (SD) rats were assessed young and middle‐aged adults for open‐field locomotor activity and reported that age did not alter the activity of Wistar males or females. However, it exerted significant effects on the open field activity levels of SD males in that their activity decreased with age and the activity of SHR males increased with age (Ferguson & Gray, 2005). Therefore, it seems that for assessing the animal behavior, strain diversity, and age difference range must be considered.

Also, previous research reported the opposed result of anxiety in old rats based on their performance in Elevated Plus Maze (EPM); the old Wistar rats were more anxious than the young ones (Boguszewski & Zagrodzka, 2002; Sotoudeh et al., 2020). There are two possible reasons: first, it seems that various methodological differences including age, strain, sex, number of the samples, and housing or testing conditions might lead to obtaining inconsistent findings in the EPM. Second, the structure of EPM is more complicated than OFT, with four narrow arms and a central platform that connected them, so rats cannot see all arms internal directly and they tend to stay in arms that they think is safer, while OFT is a simple cube and once the rats are put in the middle, it can see all‐around at the same time so it moves around and searches easier, thereby it is more possible that they show less anxiety in OFT and more in EPM.

In terms of sex difference, young female rats showed less anxiety‐like behavior than young male rats. In line with our results, Imhof et al. compared the performance of male and female Wistar rats of different ages and reported that up to 60 days of age, rats of both sexes exhibited an equal behavior; however, after 60 to 120 days of age or more, rats of both sexes exhibited statistically significant sex differences in behavior. At 90 days of age, the male rats showed high anxiety levels, whereas in females, it happened around 120 days (Imhof et al., 1993). Also, Valle (1970) reported that the number of central entrances and time increased in the female rats in OFT compared with young rats. Another study also claimed more activity in old females (Hyde & Jerussi, 1983), which confirms our data and it seems that male and female rats of similar age show different performances in the behavioral maze.

In contrast with our data, Shoji et al. (2016) tested C57BL/6J mice into the following four age groups for each behavioral test: 2–3, 4–5, 6–7, and 8–12 months of age. Their results showed the distance traveled decreased significantly with no difference in center time duration with age (Shoji et al., 2016). It seems that different age spans could constitute the reason for controversial results; in our study, only one group of young (2–3 month old) rats was compared with the old group (18–20 month‐old), but shoji et al. performed this comparison in four different age groups (2–3, 4–5, 6–7, and 8–12 months of age) with a short‐time age difference. Plus, Andersen et al. (1999) demonstrated a reduction in the frequency of social interaction in 20‐month‐old rats as compared to 4‐month‐old ones, which indicated a high level of anxiety in aged rats. Social interaction test is not specified for assessing anxiety‐like behavior in rodents and also they did not report the exact time of the test and the details of test parameters in their research (Seibenhener & Wooten, 2015). Recent studies have reported a positive or negative relationship between trait anxiety and the mPFC–amygdala connection (Modi et al., 2013; Montag et al., 2012).

Therefore, we assessed the efferent connections from the mPFC region to the right or left MeA in the young and aged rats of both sexes.

Thoroughly, we observed the bilateral connections from the mPFC subdivisions (Cg, Pl, and Il) to the right and left MeA in all groups) Tables 1, 2). First of all, some studies have confirmed the connection of Il, Pl, and Cg subregions of the mPFC to the MeA; Ghashghaei and Barbas (2002) investigated the connectivity of Il and Pl with amygdala by bidirectional tracers, horseradish peroxidase (HRP), and biotinylated dextran amines (BDA) in rhesus monkeys, and their result showed that Il and Pl axons terminated in the caudal part of amygdala strongly in BLA and MeA nuclei. In addition, Aggleton et al. (1980) injected HRP in the amygdala of nine rhesus monkeys and reported that the anterior cingulate (Cg) labeling was significantly restricted to the pyramidal cells in layer III.

Moreover, Cressman et al. (2010) injected 1% FG in the BLA nucleus of Wistar rats (P25, P45, and P90) and reported that the positively labeled cells spread in all Il, Pl, and Cg subregions.

Besides, in another study, 13 mature cats of either sex were used for the anterograde autoradiographic tracing technique in Il and Pl study and reported that fibers from Pl reached the basomedial, and central nuclei, and fibers from Il distribute to the medial and central nuclei (Room et al., 1985). These results confirm the connection of Il, Pl, and Cg with the amygdala (MeA) in our study with a difference that in our study, the labeled cells spread in different layers and are not limited to layer III (Figures 3, 4, 5).

Our results showed the OM group had a stronger mPFC–MeA top‐down connection than the YM group, while there was no significant difference between the female groups (Tables 1, 2). Importantly, some researchers have suggested that age‐related differences in functional connectivity might differ depending on task demands. For instance, in an fMRI study, in the old age prefrontal‐amygdala, functional connectivity increased in encoding positive information and decreased during the encoding of negative information (Addis et al., 2010; St. Jacques et al., 2009). However, the result of retrograde tracing studies showed that the number of Il neurons that project to the BLA decreased linearly from juvenility (P25) through adulthood, while Pl‐BLA neurons exhibited a delayed pruning pattern, remaining stable from P25 to late adolescence (P45) before sharply decreasing in number to reach adult levels at P90 in male Wistar rats (Cressman et al., 2010). Zimmermann et al. (2019) used anterograde tracing and found that fibers in the BLA which originated from the mPFC (Pl+Il) maintained a similar density from P25 to P45 and then pruned from late adolescence through adulthood (Zimmermann et al., 2019). Another study that evaluated a broader window of development (six‐time points from P10 to P80) reported a massive mPFC–BLA fiber proliferation between P10 and P30 and confirmed a modest decrease later in adolescence between P45 and adulthood (Arruda‐Carvalho et al., 2017). There is no previous study regarding the mPFC–MeA connection, and it seems that these differences in the retrograde tracing study can be related to two possible reasons; first, another research focused on the BLA, while our study was on the MeA. Second, the age span in the previous study (P10, P30, and P45) was different from our study (3‐month‐old and 18–20‐month‐old). Further, fMRI studies insist on the total connection of the PFC–amygdala connectivity, not mPFC–MeA, and distinguish it by the type of emotion and connectivity. Also, we have to consider a limitation of using Fluoro‐Gold, it can diffuse from the site of injection to the neighbor sites and lead to nonspecific labeling of neurons in the region of interest (Dickstein et al., 2007)

Regarding the relationship between trait anxiety and the mPFC–amygdala connection in both sexes; in the comparison between the two male groups, the old group had less anxiety and a stronger mPFC–MeA connection, but in the female rats, there was no significant difference in anxiety trait and also in the mPFC–MeA connection. A previous study injected FG in the BLA of young adult (8–10 weeks old) Sprague Dawley rats and concluded that Fluoro‐Gold‐positive layer II/III neurons in the Il region of high fear and low fear male differed in the male rats, but not female rats (Gruene et al., 2015). It is in accordance with the result of male groups; less anxiety in OFT coincides with a stronger mPFC–MeA connection in old rats. Also, previous studies claimed a negative relationship between trait anxiety and amygdala–mPFC connection (Modi et al., 2013; Montag et al., 2012). Overall, female animals have a strong mPFC–MeA connection at both ages with no different anxiety behavior.

In terms of lateralization, in the present study, there was no lateralization difference in the mPFC–MeA connection between old groups, but in both young groups, the right MeA had a stronger connection with bilateral mPFC subdivisions than the left MeA. Kawachi et al. (2002) in a human PET study in 63‐year‐old individuals (male and female) reported no sex‐related differences in absolute activity levels in the amygdala, while Kilpatrick et al. (2006) reported that the right amygdala was associated with greater functional connectivity in men and left amygdala in women. Most of the previous tracing studies were done on large animals such as monkeys, and there was no previous study that focused on behavior and connection of the brain areas. In addition, the MeA connections have not been completely evaluated yet.

Besides, we should point out some limitations in the current study: (1) the number of aged rats was limited in our animal lab. (2) The use of more than one behavioral test, which is also useful in making more accurate judgments, was not used. (3) Neuropeptide double staining which regulates the anxiety process in the area of interest and indeed gets to a more satisfactory conclusion was not possible.

In conclusion, the present study indicates that old individuals, predominantly male ones, have less anxiety‐like behavior and stronger mPFC–MeA connection than the young animals. In comparison between young groups, young female rats are less anxious and have a stronger connection to the mPFC–MeA than males of a similar age. Also, in male groups, there was a negative correlation between the anxiety level and the mPFC–MeA connection. In this manner, it seems that the stronger connection of mPFC–MeA could partly be the reason for less anxiety probably by regulating the process and expression of anxiety, although more studies are necessary in this regard.

## CONFLICT OF INTEREST

The authors declare no conflict of interest.

## AUTHOR CONTRIBUTIONS

**Narges Sotoudeh**: conceptualization, formal analysis, investigation, methodology, software, writing – original draft. **Mohammad R. Namavar**: conceptualization, data curation, formal analysis, funding acquisition, project administration, resources, software, supervision, validation, visualization, writing ‐ review & editing. **Asadollah Zarifkar**: conceptualization, project administration, writing – review, and editing. **Farshid Bagheri**: investigation, methodology.

## Data Availability

The data that support the findings of this study are available on request from the corresponding author. The data are not publicly available due to privacy or ethical restriction.

## References

[brb32616-bib-0001] Addis, D. R. , Leclerc, C. M. , Muscatell, K. A. , & Kensinger, E. A. (2010). There are age‐related changes in neural connectivity during the encoding of positive, but not negative, information. Cortex, 46(4), 425–433. 10.1016/j.cortex.2009.04.011 19555933 PMC2826522

[brb32616-bib-0002] Aggleton, J. , Burton, M. , & Passingham, R. (1980). Cortical and subcortical afferents to the amygdala of the rhesus monkey (*Macaca mulatta*). Brain Research, 190(2), 347–368. 10.1016/0006-8993(80)90279-6 6768425

[brb32616-bib-0003] Andersen, M. B. , Zimmer, J. , & Sams‐Dodd, F. (1999). Specific behavioral effects related to age and cerebral ischemia in rats. Pharmacology Biochemistry and Behavior, 62(4), 673–682. 10.1016/s0091-3057(98)00204-4 10208372

[brb32616-bib-0004] Arruda‐Carvalho, M. , Wu, W.‐C. , Cummings, K. A. , & Clem, R. L. (2017). Optogenetic examination of prefrontal‐amygdala synaptic development. Journal of Neuroscience, 37(11), 2976–2985. 10.1523/JNEUROSCI.3097-16.2017 28193691 PMC5354336

[brb32616-bib-0005] Baas, D. , Aleman, A. , & Kahn, R. S. (2004). Lateralization of amygdala activation: A systematic review of functional neuroimaging studies. Brain Research Reviews, 45(2), 96–103. 10.1016/j.brainresrev.2004.02.004 15145620

[brb32616-bib-0006] Banks, S. J. , Eddy, K. T. , Angstadt, M. , Nathan, P. J. , & Phan, K. L. (2007). Amygdala–frontal connectivity during emotion regulation. Social Cognitive and Affective Neuroscience, 2(4), 303–312. 10.1093/scan/nsm029 18985136 PMC2566753

[brb32616-bib-0007] Baur, V. , Hänggi, J. , & Jäncke, L. (2012). Volumetric associations between uncinate fasciculus, amygdala, and trait anxiety. BMC Neuroscience, 13(1), 4. 10.1186/1471-2202-13-4 22217209 PMC3398321

[brb32616-bib-0008] Bishop, S. J. (2007). Neurocognitive mechanisms of anxiety: An integrative account. Trends in Cognitive Sciences, 11(7), 307–316. 10.1016/j.tics.2007.05.008 17553730

[brb32616-bib-0009] Boguszewski, P. , & Zagrodzka, J. (2002). Emotional changes related to age in rats—A behavioral analysis. Behavioural Brain Research, 133(2), 323–332. 12110466 10.1016/s0166-4328(02)00018-9

[brb32616-bib-0010] Brennan, P. A. , & Zufall, F. (2006). Pheromonal communication in vertebrates. Nature, 444(7117), 308–315. 10.1038/nature05404 17108955

[brb32616-bib-0011] Cádiz‐Moretti, B. , Otero‐García, M. , Martínez‐García, F. , & Lanuza, E. (2016). Afferent projections to the different medial amygdala subdivisions: A retrograde tracing study in the mouse. Brain Structure and Function, 221(2), 1033–1065. 10.1007/s00429-014-0954-y 25503449

[brb32616-bib-0012] Cassell, M. , & Wright, D. (1986). Topography of projections from the medial prefrontal cortex to the amygdala in the rat. Brain Research Bulletin, 17(3), 321–333. 10.1016/0361-9230(86)90237-6 2429740

[brb32616-bib-0013] Cressman, V. L. , Balaban, J. , Steinfeld, S. , Shemyakin, A. , Graham, P. , Parisot, N. , & Moore, H. (2010). Prefrontal cortical inputs to the basal amygdala undergo pruning during late adolescence in the rat. Journal of Comparative Neurology, 518(14), 2693–2709. 10.1002/cne.22359 20506471 PMC3377974

[brb32616-bib-0014] Dickstein, D. L. , Kabaso, D. , Rocher, A. B. , Luebke, J. I. , Wearne, S. L. , & Hof, P. R. (2007). Changes in the structural complexity of the aged brain. Aging Cell, 6(3), 275–284. 17465981 10.1111/j.1474-9726.2007.00289.x PMC2441530

[brb32616-bib-0015] Ebner, N. C. , & Fischer, H. (2014). Emotion and aging: Evidence from brain and behavior. Frontiers in Psychology, 5, 996. 25250002 10.3389/fpsyg.2014.00996 PMC4158975

[brb32616-bib-0016] Ferguson, S. A. , & Gray, E. P. (2005). Aging effects on elevated plus maze behavior in spontaneously hypertensive, Wistar–Kyoto and Sprague–Dawley male and female rats. Physiology & Behavior, 85(5), 621–628. 10.1016/j.physbeh.2005.06.009 16043200

[brb32616-bib-0017] Ghashghaei, H. , & Barbas, H. (2002). Pathways for emotion: Interactions of prefrontal and anterior temporal pathways in the amygdala of the rhesus monkey. Neuroscience, 115(4), 1261–1279. 10.1016/s0306-4522(02)00446-3 12453496

[brb32616-bib-0018] Ghashghaei, H. , Hilgetag, C. C. , & Barbas, H. (2007). Sequence of information processing for emotions based on the anatomic dialogue between prefrontal cortex and amygdala. Neuroimage, 34(3), 905–923. 10.1016/j.neuroimage.2006.09.046 17126037 PMC2045074

[brb32616-bib-0019] Grady, C. (2012). The cognitive neuroscience of ageing. Nature Reviews Neuroscience, 13(7), 491. 10.1038/nrn3256 22714020 PMC3800175

[brb32616-bib-0020] Gruene, T. M. , Roberts, E. , Thomas, V. , Ronzio, A. , & Shansky, R. M. (2015). Sex‐specific neuroanatomical correlates of fear expression in prefrontal‐amygdala circuits. Biological Psychiatry, 78(3), 186–193. 25579850 10.1016/j.biopsych.2014.11.014 PMC4449316

[brb32616-bib-0021] Hyde, J. F. , & Jerussi, T. P. (1983). Sexual dimorphism in rats with respect to locomotor activity and circling behavior. Pharmacology Biochemistry and Behavior, 18(5), 725–729. 10.1016/0091-3057(83)90014-x 6856647

[brb32616-bib-0022] Imhof, J. T. , Coelho, Z. M. , Schmitt, M. L. , Morato, G. S. , & Carobrez, A. P. (1993). Influence of gender and age on performance of rats in the elevated plus maze apparatus. Behavioural Brain Research, 56(2), 177–180. 10.1016/0166-4328(93)90036-p 8240712

[brb32616-bib-0023] Kawachi, T. , Ishii, K. , Sakamoto, S. , Matsui, M. , Mori, T. , & Sasaki, M. (2002). Gender differences in cerebral glucose metabolism: A PET study. Journal of the Neurological Sciences, 199(1‐2), 79–83. 10.1016/s0022-510x(02)00112-0 12084447

[brb32616-bib-0024] Kilpatrick, L. A. , Zald, D. H. , Pardo, J. V. , & Cahill, L. (2006). Sex‐related differences in amygdala functional connectivity during resting conditions. Neuroimage, 30(2), 452–461. 10.1016/j.neuroimage.2005.09.065 16326115

[brb32616-bib-0025] Kim, M. J. , Gee, D. G. , Loucks, R. A. , Davis, F. C. , & Whalen, P. J. (2011). Anxiety dissociates dorsal and ventral medial prefrontal cortex functional connectivity with the amygdala at rest. Cerebral Cortex, 21(7), 1667–1673. 10.1093/cercor/bhq237 21127016 PMC3116741

[brb32616-bib-0026] Kim, M. J. , & Whalen, P. J. (2009). The structural integrity of an amygdala–prefrontal pathway predicts trait anxiety. Journal of Neuroscience, 29(37), 11614–11618. 10.1523/JNEUROSCI.2335-09.2009 19759308 PMC2791525

[brb32616-bib-0027] Krettek, J. , & Price, J. (1977). Projections from the amygdaloid complex to the cerebral cortex and thalamus in the rat and cat. Journal of Comparative Neurology, 172(4), 687–722. 10.1002/cne.901720408 838895

[brb32616-bib-0028] LeDoux, J. (2012). Rethinking the emotional brain. Neuron, 73(4), 653–676. 10.1016/j.neuron.2012.02.004 22365542 PMC3625946

[brb32616-bib-0029] McLaughlin, K. A. , Sheridan, M. A. , & Lambert, H. K. (2014). Childhood adversity and neural development: Deprivation and threat as distinct dimensions of early experience. Neuroscience & Biobehavioral Reviews, 47, 578–591. 10.1016/j.neubiorev.2014.10.012 25454359 PMC4308474

[brb32616-bib-0030] Modi, S. , Trivedi, R. , Singh, K. , Kumar, P. , Rathore, R. K. , Tripathi, R. P. , & Khushu, S. (2013). Individual differences in trait anxiety are associated with white matter tract integrity in fornix and uncinate fasciculus: Preliminary evidence from a DTI based tractography study. Behavioural Brain Research, 238, 188–192. 10.1016/j.bbr.2012.10.007 23085341

[brb32616-bib-0031] Montag, C. , Reuter, M. , Weber, B. , Markett, S. , & Schoene‐Bake, J.‐C. (2012). Individual differences in trait anxiety are associated with white matter tract integrity in the left temporal lobe in healthy males but not females. Neuroscience, 217, 77–83. 10.1016/j.neuroscience.2012.05.017 22609931

[brb32616-bib-0032] Ochsner, K. N. , & Gross, J. J. (2005). The cognitive control of emotion. Trends in Cognitive Sciences, 9(5), 242–249. 10.1016/j.tics.2005.03.010 15866151

[brb32616-bib-0033] Olucha‐Bordonau, F. E. , Fortes‐Marco, L. , Otero‐García, M. , Lanuza, E. , & Martínez‐García, F. (2015). Amygdala: Structure and function. In The rat nervous system (pp. 441–490). Elsevier.

[brb32616-bib-0034] Pezawas, L. , Meyer‐Lindenberg, A. , Drabant, E. M. , Verchinski, B. A. , Munoz, K. E. , Kolachana, B. S. , Egan, M. F. , Mattay, V. S. , Hariri, A. R. , & Weinberger, D. R. (2005). 5‐HTTLPR polymorphism impacts human cingulate‐amygdala interactions: A genetic susceptibility mechanism for depression. Nature Neuroscience, 8(6), 828–834. 10.1038/nn1463 15880108

[brb32616-bib-0035] Phelps, E. A. , Delgado, M. R. , Nearing, K. I. , & LeDoux, J. E. (2004). Extinction learning in humans: Role of the amygdala and vmPFC. Neuron, 43(6), 897–905. 10.1016/j.neuron.2004.08.042 15363399

[brb32616-bib-0036] Phelps, E. A. , & LeDoux, J. E. (2005). Contributions of the amygdala to emotion processing: From animal models to human behavior. Neuron, 48(2), 175–187. 10.1016/j.neuron.2005.09.025 16242399

[brb32616-bib-0037] Qi, Y. , Namavar, M. R. , Iqbal, J. , Oldfield, B. J. , & Clarke, I. J. (2009). Characterization of the projections to the hypothalamic paraventricular and periventricular nuclei in the female sheep brain, using retrograde tracing and immunohistochemistry. Neuroendocrinology, 90(1), 31–53. 10.1159/000221304 19478473

[brb32616-bib-0038] Quirk, G. J. , & Beer, J. S. (2006). Prefrontal involvement in the regulation of emotion: Convergence of rat and human studies. Current Opinion in Neurobiology, 16(6), 723–727. 10.1016/j.conb.2006.07.004 17084617

[brb32616-bib-0039] Room, P. , Russchen, F. T. , Groenewegen, H. J. , & Lohman, A. H. (1985). Efferent connections of the prelimbic (area 32) and the infralimbic (area 25) cortices: An anterograde tracing study in the cat. Journal of Comparative Neurology, 242(1), 40–55. 10.1002/cne.902420104 4078047

[brb32616-bib-0040] Samson, R. D. , & Barnes, C. A. (2013). Impact of aging brain circuits on cognition. European Journal of Neuroscience, 37(12), 1903–1915. 23773059 10.1111/ejn.12183 PMC3694726

[brb32616-bib-0041] Seibenhener, M. L. , & Wooten, M. C. (2015). Use of the open field maze to measure locomotor and anxiety‐like behavior in mice. Journal of Visualized Experiments, 96, e52434. 10.3791/52434 PMC4354627 25742564

[brb32616-bib-0042] Sestakova, N. , Puzserova, A. , Kluknavsky, M. , & Bernatova, I. (2013). Determination of motor activity and anxiety‐related behaviour in rodents: Methodological aspects and role of nitric oxide. Interdisciplinary Toxicology, 6(3), 126–135. 24678249 10.2478/intox-2013-0020 PMC3967438

[brb32616-bib-0043] Shoji, H. , & Miyakawa, T. (2019). Age‐related behavioral changes from young to old age in male mice of a C57 BL/6J strain maintained under a genetic stability program. Neuropsychopharmacology Reports, 39(2), 100–118. 10.1002/npr2.12052 30816023 PMC7292274

[brb32616-bib-0044] Shoji, H. , Takao, K. , Hattori, S. , & Miyakawa, T. (2016). Age‐related changes in behavior in C57BL/6J mice from young adulthood to middle age. Molecular Brain, 9(1), 11. 10.1186/s13041-016-0191-9 26822304 PMC4730600

[brb32616-bib-0045] Sotoudeh, N. , Namavar, M. , Zarifkar, A. , & Heidarzadegan, A. (2020). Age‐dependent changes in the medial prefrontal cortex and medial amygdala structure, and elevated plus‐maze performance in the healthy male Wistar rats. IBRO Reports, 9, 183–194. 32885088 10.1016/j.ibror.2020.08.002 PMC7452646

[brb32616-bib-0046] St Jacques, P. L. , Dolcos, F. , & Cabeza, R. (2009). Effects of aging on functional connectivity of the amygdala for subsequent memory of negative pictures: A network analysis of functional magnetic resonance imaging data. Psychological Science, 20(1), 74–84. 10.1111/j.1467-9280.2008.02258.x 19152542 PMC3633516

[brb32616-bib-0047] Torras‐Garcia, M. , Costa‐Miserachs, D. , Coll‐Andreu, M. , & Portell‐Cortés, I. (2005). Decreased anxiety levels related to aging. Experimental Brain Research, 164(2), 177–184. 15856210 10.1007/s00221-005-2240-y

[brb32616-bib-0048] Vafaee, F. , Zarifkar, A. , Emamghoreishi, M. , Namavar, M. R. , Shahpari, M. , & Zarifkar, A. H. (2018). Effect of recombinant insulin‐like growth factor‐2 injected into the hippocampus on memory impairment following hippocampal intracerebral hemorrhage in rats. Galen Med J, 7, e1353. 10.22086/gmj.v0i0.1353 34466449 PMC8344085

[brb32616-bib-0049] Valle, F. P. (1970). Effects of strain, sex, and illumination on open‐field behavior of rats. The American Journal of Psychology, 83(1), 103–111. PMID 5465190. 5465190

[brb32616-bib-0050] Wager, T. D. , Davidson, M. L. , Hughes, B. L. , Lindquist, M. A. , & Ochsner, K. N. (2008). Prefrontal‐subcortical pathways mediating successful emotion regulation. Neuron, 59(6), 1037–1050. 10.1016/j.neuron.2008.09.006 18817740 PMC2742320

[brb32616-bib-0051] Wager, T. D. , Phan, K. L. , Liberzon, I. , & Taylor, S. F. (2003). Valence, gender, and lateralization of functional brain anatomy in emotion: A meta‐analysis of findings from neuroimaging. Neuroimage, 19(3), 513–531. 10.1016/s1053-8119(03)00078-8 12880784

[brb32616-bib-0052] Zimmermann, K. S. , Richardson, R. , & Baker, K. D. (2019). Maturational changes in prefrontal and amygdala circuits in adolescence: Implications for understanding fear inhibition during a vulnerable period of development. Brain Sciences, 9(3), 65. 10.3390/brainsci9030065 PMC6468701 30889864

